# Significance of Hypocapnia in the Risk Assessment of Patients with Pulmonary Hypertension

**DOI:** 10.3390/jcm12196307

**Published:** 2023-09-30

**Authors:** Maria Aetou, Lora Wahab, Michael Dreher, Ayham Daher

**Affiliations:** Department of Pneumology and Intensive Care Medicine, University Hospital RWTH Aachen, 52074 Aachen, Germany; maetou@ukaachen.de (M.A.); lwahab@ukaachen.de (L.W.); adaher@ukaachen.de (A.D.)

**Keywords:** partial pressure of carbon dioxide, hypocapnia, hyperventilation, pulmonary hypertension

## Abstract

Blood gas analysis is part of the diagnostic work−up for pulmonary hypertension (PH). Although some studies have found that the partial pressure of carbon dioxide (PaCO_2_) is an independent marker of mortality in individuals with pulmonary arterial hypertension (PH Group 1), there is a lack of data regarding the significance of PaCO_2_ in individuals with different types of PH based on the new 2022 definitions. Therefore, this study analyzed data from 157 individuals who were undergoing PH work−up, including right heart catheterization, using PH definitions from the 2022 European Society of Cardiology/European Respiratory Society guidelines. At diagnosis, N−terminal pro−B−type natriuretic peptide (NT−pro−BNP) levels were significantly higher, but the time−course of NT−pro−BNP levels during treatment was significantly more favorable in individuals with pulmonary arterial hypertension (PH Group 1) who did versus did not have hypocapnia (*p* = 0.026 and *p* = 0.017, respectively). These differences based on the presence of hypocapnia were not seen in individuals with PH Groups 2, 3, or 4. In conclusion, using the new definition of PH, hypocapnia may correlate with worse risk stratification at diagnosis in individuals with pulmonary arterial hypertension. However, hypocapnic individuals with pulmonary arterial hypertension may benefit more from disease−specific therapy than those without hypocapnia.

## 1. Introduction

Pulmonary hypertension (PH) is a complex and serious disease that is commonly seen by physicians across a range of specialties [1]. Furthermore, PH is a global health topic of considerable importance, and current estimates suggest that the worldwide prevalence of pulmonary hypertension is about 1%, with the rate increasing to 10% in people aged > 65 years [2]. Data show higher rates of PH as age increases, and highlight the relevance of an aging population [3]. PH was initially defined as a resting mean pulmonary arterial pressure (mPAP) of ≥ 25 mmHg, measured using right heart catheterization in the supine position [4]. Currently, PH is classified into five different groups based on presentation and underlying etiology [5]:

- PH Group 1—Pulmonary arterial hypertension (PAH);
- PH Group 2—Pulmonary hypertension associated with left heart disease (PH−LHD);
- PH Group 3—Pulmonary hypertension associated with lung diseases and/or hypoxia; pulmonary hypertension associated with chronic lung disease (PH−CLD);
- PH Group 4—Chronic thromboembolic pulmonary hypertension (CTEPH);
- PH Group 5—Pulmonary hypertension with unclear and/or multifactorial mechanisms.

Significant progress has been made in the detection and treatment of PH over recent years. At the sixth World Symposium on Pulmonary Hypertension in 2018, it was proposed that the mPAP threshold used to define PH should be lowered from ≥25 mmHg to >20 mmHg [6]. The rationale for this change was that the ≥25 mmHg threshold was arbitrary, whereas the revised threshold was based on scientific evidence [7]. The threshold mPAP >20 mmHg has been shown to be significantly associated with increased risks for progression to overt PH, hospitalizations, and mortality [8,9,10]. In the 2022 European Society of Cardiology (ESC)/European Respiratory Society (ERS) guidelines for pulmonary hypertension [11], the hemodynamic definition of PH has been officially updated using the new mPAP threshold >20 mmHg, but the threshold for pulmonary vascular resistance (PVR) was also updated based on current evidence, and it was stated that the upper limit of normal PVR and the lowest prognostically relevant threshold for PVR is 2 Wood units (WU) [11]. Furthermore, the new ESC/ERS guidelines gave an update of the therapy algorithm focusing on risk stratification and the importance of combination therapies at the right time [11]. These developments highlight the complexity of PH and the fact that its treatment requires a multifaceted, holistic, and multidisciplinary approach [12].

Given that mPAP above the upper limit of normal (>20 mmHg) but below 25 mmHg is associated with increased risk of morbidity and mortality compared with a normal mPAP [8,9,10,13,14,15], early identification of individuals who have mPAP between 20 and 25 mmHg is important to enable close monitoring and timely treatment initiation once clinically indicated, even if PAH−specific medications have not been widely approved for individuals who have a mPAP within this range [14]. However, some subgroups of individuals might be more likely to benefit from early treatment, including those with systemic sclerosis [16] or mutations associated with PH, who may need PAH−specific treatments early in their disease course.

In general, three factors are used for risk stratification in PH: functional class, 6 min walk distance, and B−type natriuretic peptide levels [12,17,18]. But blood gas analysis is also part of the approach to the management of PH, and the results of blood gas analysis are often not normal in these patients [19]. However, severe hypocapnia is more common than severe hypoxemia in patients with PH [20], and significant hypocapnia, defined as an arterial partial pressure of carbon dioxide (PaCO_2_) of <32 mmHg, has also been shown to be an independent predictor of mortality in individuals with idiopathic pulmonary arterial hypertension (iPAH) [19,21]. It has also been reported that measuring PaCO_2_ at diagnosis and during follow−up in people with PAH provided independent prognostic information and has the potential to improve current risk assessment strategies [22]. It has been hypothesized that the hypocapnia seen in patients with PH is due to hyperventilation, which is essentially related to increased chemosensitivity as a mechanism to compensate for underlying hypoxemia [19,20]. Nevertheless, there is no evidence to support this hypothesis, and hypocapnia could have other pathophysiological associations with PH, such as relationships with pathological changes in the pulmonary arteries and surrounding tissues. These relationships could have prognostic implications and interact with treatments for PH. In other words, peripheral hypocapnia might be related to pathological abnormalities in the pulmonary vasculature, which would make PaCO_2_ an important parameter in risk stratification of patients with PH.

Since hypocapnia may have a role to play in the risk stratification of individuals with PH, it is important to understand the relationships between PaCO_2_ and clinical features for each type of PH, other risk variables, and follow−up patterns during treatment. However, there is currently a lack of data about the significance of PaCO_2_ in different PH groups and when using the new hemodynamic definition of PH. Therefore, this study evaluated the importance of hypocapnia in individuals with different types of PH who were diagnosed using the new PH definition, and it investigated correlations between hypocapnia and disease course during follow−up.

## 2. Materials and Methods

### 2.1. Study Design

This retrospective study was conducted at the University Hospital Aachen of RWTH Aachen University. The study protocol was approved by the local ethics committee (The Independent Ethics Committee at the RWTH Aachen Faculty of Medicine, EK 041/21), and all study procedures were performed in accordance with the ethical standards laid down in the Declaration of Helsinki and its latest revision. Due to the retrospective study design, the requirement for informed consent to participate has been waived by the local ethics committee.

### 2.2. Participants

All patients admitted to our institution due to undergo right heart catheterization between January 2014 and April 2023 were retrospectively screened for eligibility. Patients were included only if the full results of hemodynamic measurements of right heart catheterization, pulmonary function tests (PFTs) including blood gas analysis (BGA), and an adequate risk stratification including NT−pro−BNP measurement were available. Individuals with confirmed PH based on the new ESC/ERS definition (i.e., mPAP > 20 mmHg and PVR > 2 WU) [11] were included in this study. Those with any type of PH were eligible, but the small number of individuals with Group 5 PH meant that no specific analysis was further performed in this subgroup.

### 2.3. Data Collection and Assessments

Clinical patient−related data and pulmonary and laboratory parameters were recorded anonymously in statistical spreadsheets. Patient data were retrieved from the patient data management system (CGM MEDICO; CompuGroup Medical Clinical Europe GmbH, Koblenz, Germany). Baseline information recorded included demographic data (i.e., age, height, weight, sex, smoking status), comorbidities, medication, results of right heart catheterization (RHC) (including PH group), PFT results, and BGA from the arterialized earlobe. Samples for arterial BGA were taken from the arterialized earlobes of all patients while breathing room air without supplemental oxygen (ABL 800 flex; Radiometer, Copenhagen, Denmark). In addition, data on the following were recorded at baseline, after 3–6 months, and after 7–12 months: blood results (hemoglobin, N−terminal pro−B−type natriuretic peptide [NT−pro−BNP], creatinine, alanine aminotransferase [ALT]; aspartate amino transaminase [AST]), World Health Organization (WHO) functional classification, and the 6 min walk distance (6MWD). Participants were divided into two groups based on their PaCO_2_ value from BGA performed at the time of PH diagnosis (<35 mmHg (i.e., hypocapnia) versus ≥35 mmHg (i.e., no hypocapnia)).

### 2.4. Statistical Analysis

The programming language Python 3.9.13, with statsmodels library version 0.13.2 and SciPy library version 1.9.1, was used for all statistical analysis. Jupyter Notebook Version 6.4.12 was used for data exploration and visualization.

Mean and standard deviation values or frequency distribution were summarized for all the demographic data, and for variables of interest for individuals with or without hypocapnia. A simplified one−year mortality risk assessment tool was used to predict mortality during follow−up (using the variables NT−pro−BNP, 6MWD, and WHO functional classification). Changes in risk assessment variables at each follow−up were calculated and mean and standard deviation values for NT−pro−BNP and the 6MWD were reported. The Kruskal–Wallis test was used to compare changes in NT−pro−BNP over time in individuals with versus without hypocapnia. The Mann–Whitney U−test was used to compare the NT−pro−BNP variable distribution at each timepoint in the subgroups with or without hypocapnia. Differences between the subgroups with and without hypocapnia were examined for each variable of interest at baseline and follow−up using a permutation test (one−tailed) for two independent samples with 10,000 random permutations. Significance level was set at α = 0.05.

The 6MWD is often used for the calculation of cohort sizes in drug trials in patients with PH. Therefore, assuming an effect size of 38.4 m with standard deviation at baseline of 77 m [23], with a one−sided significance of alpha = 0.05 and a power of 0.8, a sample size of 50 patients per group was estimated to be required.

Data were stratified according to the clinical classification of PH from the 2022 ESC/ERS guidelines for the diagnosis and treatment of PH [11]. The family−wise error rate was accounted for using the Holm–Šídák correction method. There was no imputation of missing values.

## 3. Results

### 3.1. Participants

A total of 157 individuals were included, of whom 30% had Group 1 PH, 29% had Group 2 PH, 28% had Group 3 PH, and 10% had Group 4 PH; several comorbidities were common (Table 1).

### 3.2. Hypocapnia and Its Correlates

In total, 62 patients with PH (39%) had hypocapnia at the time of PH diagnosis. Considering the whole cohort, individuals with versus without hypocapnia tended to have higher NT−pro−BNP at baseline (*p* = 0.089) and at the first follow−up (*p* = 0.065), but NT−pro−BNP levels were similar in the two subgroups at the second follow−up (Table 2).

In individuals with PAH (PH Group 1), levels of NT−pro−BNP were significantly higher in those with versus without hypocapnia (4529 ± 5646 vs. 1380 ± 1429, *p* = 0.026) (Table 3). PAH patients with and without hypocapnia were comparable regarding comorbidities, pulmonary functions tests, and hemodynamic variables including cardiac output (CO), mPAP, PVR, and pulmonary arterial wedge pressure (PAWP). There was no significant difference in NT−pro−BNP between those with and without hypocapnia in individuals with PH Group 2 and 3 (*p* > 0.05). For individuals with PH Group 4, NT−pro−BNP levels were at time of diagnosis slightly, but not significantly, lower in those with versus without hypocapnia (*p* = 0.21) (Table 3).

**Figure 1:**
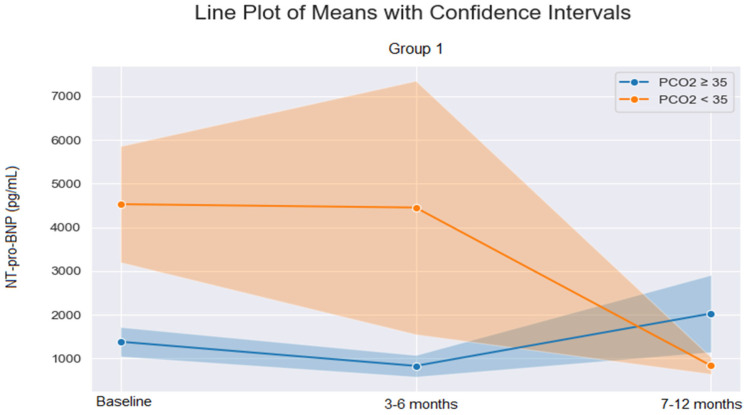
Change in N−terminal pro−B−type natriuretic peptide (NT−pro−BNP) values over time in individuals with pulmonary artery hypertension (PH Group 1) in individuals with versus without hypocapnia (PCO_2_ <35 vs. ≥35 mmHg); shaded areas on either side of the lines indicate the confidence intervals.

Nearly all (46/48) individuals with PAH (PH Group 1) were treated with PAH−specific therapy. This included phosphodiesterase−5 inhibitors (PDE−5i), endothelin receptor antagonists (ERAs), prostanoids, and highly dosed calcium channel blockers (CCBs) by proven reversibility. The reduction in NT−pro−BNP levels during treatment was significantly greater in individuals with versus without hypocapnia (*p* = 0.017) (Figure 1); there was no difference in the effects of treatment on NT−pro−BNP in the other PH groups (all groups *p* > 0.05).

At the time of diagnosis, the 6MWD and WHO classification for individuals with PAH (PH Group 1) did not differ significantly between those with or without hypocapnia (*p* > 0.05).

## 4. Discussion

The results of this study showed that hypocapnia was associated with higher baseline levels of NT−pro−BNP in individuals with group 1 PH (PAH) diagnosed using the new hemodynamic definition. In addition, individuals with PAH who had hypocapnia showed greater improvements in NT−pro−BNP during PAH−specific treatment than those without hypocapnia. 

Baseline PaCO_2_ at rest has been reported to influence survival in people with idiopathic PAH [21]. Survival rates were lower in those with a baseline PaCO_2_ (at first diagnosis) of <32 mmHg [21]. However, in that study, hypocapnia at rest and during exercise correlated with low cardiac output, low peak oxygen uptake, and reduced ventilatory efficacy, which may have been confounding factors. In our study, patients with and without hypocapnia were comparable regarding hemodynamic variables including cardiac output, exercise endurance represented in the 6MWD, and the WHO functional class. Despite this, individuals with baseline hypocapnia (PaCO_2_ <35 mmHg at first diagnosis) had higher NT−pro−BNP levels than those without hypocapnia, except for those with PH Group 4, which represents a specific phenotype of PH (the between−group difference was statistically significant in patients with PH Group 1). People with thromboembolic pulmonary disease (PH Group 4) are often hypocapnic, irrespective of the presence or absence of PH [24]. In this subgroup of people with PH, it could be speculated that hypocapnia indicates respiratory compensation and may be related to better prognosis. Our data support this because individuals with PH Group 4 had lower NT−pro−BNP when they had hypocapnia at baseline.

Regarding patients with PH Group 1 (PAH), we are not aware of any evidence to support the hypothesis of increased respiratory drive (hyperventilation due to an increased chemosensitivity of the respiratory center) to explain hypocapnia. Therefore, other pathophysiological explanations should be considered, which may be supported by our results. An analysis of postcapillary blood gases in a small retrospective study showed that patients with PAH had significantly lower PaCO_2_ values in blood gases derived from the pulmonary artery than patients with PH in Groups 2–5, and this difference becomes much more pronounced in postcapillary gases (i.e., with more pulmonary passage of blood) [25]. This implies that hypocapnia is a very consistent feature of PH Group 1 (PAH) and could have other explanations, perhaps due to specific features and pathophysiological changes in the pulmonary artery that play a central role in PAH, such as endothelial dysfunction. Our results may support this because patients with hypocapnia had higher NT−pro−BNP levels, which may indicate more severe vasculopathy and greater right ventricular stress. Logically, hypocapnia means that most of the blood passing through the pulmonary circulation is flowing through areas with good ventilation. Increased hyperventilation due to hypoxemia would be an explanation for hypocapnia. There are two other possible explanations: an increased amount of blood passing through the pulmonary vasculature and increased transport of CO_2_ into the alveoli. An increased amount of blood passing through the pulmonary vasculature could be due to inappropriately increased energy output from the right ventricle to overcome the uncoupling between the right ventricle and pulmonary artery, which could also explain the higher NT−pro−BNP values in patients with hypocapnia. However, endothelial hypertrophy in still open capillaries may facilitate CO_2_ transport into the alveoli, providing another possible explanation for both hypocapnia and increased stress on the right ventricle resulting in increased NT−pro−BNP levels.

High NT−pro−BNP is known to be a biomarker for severe disease in individuals with PAH [26], and the current findings might indicate that PaCO_2_ could be a useful marker of risk at first diagnosis in individuals with PAH. However, future studies with larger sample sizes are needed to investigate this further.

Along with the NT−pro−BNP level at diagnosis, levels during follow−up might be just as important in terms of prognosis and allow more precise risk stratification [27]. Elevated plasma NT−pro−BNP levels are associated with increased mortality in patients with PAH, but a fall in NT−pro−BNP levels after therapy is associated with improved survival [28,29]. Interestingly, in our study, individuals with PAH who had hypocapnia at baseline showed greater improvements of NT−pro−BNP during follow−up than similar individuals without hypocapnia. This might simply indicate greater effectiveness of PAH−specific treatment, meaning that, while hypocapnic individuals with PAH might have more severe disease at presentation, this phenotype might actually benefit more from PAH−specific therapy. One can speculate that hypocapnia is associated with more pulmonary circulation changes that may partially improve with therapy, whereas patients without hypocapnia may have more chronic refractory abnormalities. Also, hypocapnic individuals with PAH might be a subgroup who have severe illness but also have better reserves (reflected by the ability to hyperventilate) and may therefore benefit better from PAH−specific treatments. Importantly, PAH patients with and without hypocapnia in our study were comparable regarding some significant confounders (including hemodynamic variables such as cardiac output, exercise endurance (6MWD), and the WHO functional class), which could explain why hypocapnia at baseline had favorable effects at follow−up compared with previous studies.

Independent of underlying disease, the development of PH is associated with clinical deterioration and a substantial increase in mortality risk. Global population ageing and increased life expectancy will increase the number of cases presenting to the medical system with an illness that was, until relatively recently, not widely understood, suspected, diagnosed, and treated. Efforts to refine evaluation algorithms therefore continue, and several authors have suggested that using a combination of parameters may better identify those at high risk of PH, perhaps due to the limitations of currently available tools [30]. Of these parameters, we have shown that PaCO_2_ can and should be considered in the diagnosis of PH and during patient follow−up. It represents an easy−to−use tool to help identify individuals who should be monitored closely and for whom early therapy should be considered. It will also be interesting to determine whether hypocapnia should be considered in the treatment decision making process for PH (e.g., in those with “borderline” PH).

## 5. Conclusions

In individuals with PAH diagnosed using the new hemodynamic criteria, hypocapnia was a marker of disease severity at baseline, but was associated with better response to PAH−specific therapy. It therefore seems important to include determination of PaCO_2_ to detect hypocapnia as part of the assessment and follow−up of individuals with PAH.

## Figures and Tables

**Table 1:** Participant characteristics at baseline.

Characteristics	Participants (*n* = 157)
Age, years	70 ± 11
Male sex, *n* (%)	65 (41)
**Smoking status**	
Smoker, *n* (%)	23 (15)
Ex−smoker, *n* (%)	55 (35)
Smoking pack−years	40
**Pulmonary hypertension group ^1^, *n* (%)**	
1	48 (30)
2	45 (29)
3	44 (28)
4	16 (10)
**Comorbidities, *n* (%)**	
Heart failure with reduced ejection fraction	5 (3)
Heart failure with mid−range ejection fraction	21 (13)
Heart failure with preserved ejection fraction	106 (68)
Arterial hypertension	113 (72)
Atrial fibrillation	50 (32)
Coronary heart disease	42 (27)
Valvular cardiomyopathy	8 (5)
Chronic obstructive pulmonary disease	54 (35)
Asthma	13 (8)
Interstitial lung disease	32 (20)
Diabetes mellitus	55 (35)
Systemic sclerosis	13 (8)
Connective tissue disease	15 (10)
Obstructive sleep apnea syndrome	30 (19)
Pulmonary embolism	52 (33)
History of lung cancer	6 (4)
Dyslipidemia	77 (49)
Obesity	52 (33)
Chronic renal insufficiency	53 (34)
**Medications *n* (%)**	
β−blockers	73 (47)
Angiotensin converting enzyme inhibitors	50 (32)
Angiotensin receptor blockers	33 (21)
Calcium channel blockers	28 (18)
Thiazide diuretics	36 (23)
Mineralocorticoid receptor antagonist	44 (28)
Long−acting β−agonists	64 (41)
Long−acting muscarinic antagonists	62 (40)
Inhaled corticosteroids	37 (24)
Phosphodiesterase−5 inhibitor	61 (39)
Riociguat	14 (9)
Endothelin receptor antagonists	37 (24)
Prostanoids	5 (3)
Highly dosed calcium channel blocker by proven reversibility	4 (3)
**Laboratory tests**	
Hemoglobin, g/dL	13.27 ± 2.08
Creatinine, mg/dL	1.20 ± 0.72
Aspartate aminotransferase, U/L	29.4 ± 14.0
Alanine aminotransferase, U/L	26.7 ± 21.2
NT−pro−BNP, pg/mL	3428.8 ± 5079.7

Values are mean ± standard deviation or number of participants (%). ^1^ Based on the current European Society of Cardiology/European Respiratory Society guidelines. NT−pro−BNP, N−terminal pro−B−type natriuretic peptide.

**Table 2:** Characteristics of participants with versus without hypocapnia for individuals with pulmonary hypertension of all groups.

Characteristics	Pulmonary Hypertension		
	With Hypocapnia (*n* = 62)	Without Hypocapnia (*n* = 95)	*p*-Value
Age, years	69.7 ± 9.5	70.2 ± 12.1	0.998
Hemoglobin, g/dL	13.31 ± 2.3	13.29 ± 1.95	0.998
Creatinine, mg/dL	1.23 ± 0.57	1.19 ± 0.81	0.994
HFpEF, *n* (%)	37 (24)	67 (43)	
Arterial hypertension, *n* (%)	39 (25)	71 (45)	
Atrial fibrillation, *n* (%)	14 (9)	35 (22)	
Coronary heart disease, *n* (%)	21 (13)	19 (12)	
COPD, *n* (%)	17 (11)	36 (23)	
Interstitial lung disease, *n* (%)	11 (7)	17 (11)	
Diabetes mellitus, *n* (%)	20 (13)	33 (21)	
Connective tissue disease, *n* (%)	4 (3)	11 (7)	
Systemic sclerosis, *n* (%)	9 (6)	4 (3)	
Dyslipidemia, *n* (%)	31 (20)	44 (28)	
Chronic renal insufficiency, *n* (%)	20 (123)	33 (21)	
**At diagnosis/baseline**			
Right atrial pressure	9.5 ± 4.7	10.3 ± 4.9	0.145
Cardiac index	2.5 ± 0.7	2.7 ± 1.0	0.096
Stroke volume index	0.034 ± 0.011	0.036 ± 0.016	0.217
Venous oxygen saturation	62.6 ± 10.3	60.4 ± 9.0	0.12
WHO functional class	3.1 ± 0.7	2.9 ± 0.7	0.188
NT−pro−BNP	4252.6 ± 5404.7	2926.7 ± 4845.6	0.089
**At 3− to 6−month follow−up**			
WHO functional class	2.4 ± 0.8	2.4 ± 0.8	0.444
NT−pro−BNP	3342.3 ± 10,333.9	1173.1 ± 1729.7	0.065
**At 7− to 12−month follow−up**			
WHO functional class	2.4 ± 1.0	2.5 ± 0.9	0.512
NT−pro−BNP	1427.0 ± 1975.7	1634.8 ± 2571.9	0.374

Values are mean ± standard deviation or number of participants (%). COPD, chronic obstructive pulmonary disease; HFpEF, heart failure with preserved ejection fraction; NT−pro−BNP, N−terminal pro−B−type natriuretic peptide; WHO, World Health Organization.

**Table 3:** NT−pro−BNP values of participants with versus without hypocapnia for individuals within each pulmonary hypertension group.

NT−Pro−BNP	Pulmonary Artery Hypertension		
	With Hypocapnia	Without Hypocapnia	*p*-Value
**At diagnosis/baseline**			
PH Group 1	4529 ± 5646	1380 ± 1429	0.026
PH Group 2	5711 ± 6363	3220 ± 4393	0.31
PH Group 3	3617 ± 5433	1917 ± 3442	0.31
PH Group 4	1433 ± 1550	12,821 ± 11,093	0.21
**At 3− to 6−month follow−up**			
PH Group 1	4452 ± 13,623	827 ± 963	0.33
PH Group 2	3802 ± 3819	2545 ± 3723	0.51
PH Group 3	1591 ± 1506	847 ± 953	0.36
PH Group 4	873 ± 824	2750 ± 3524	0.37
**At 7− to 12−month follow−up**			
PH Group 1	832 ± 810	2026 ± 3428	0.23
PH Group 2	3795 ± 4426	2865 ± 1579	0.64
PH Group 3	3288 ± 585	645 ± 786	0.07
PH Group 4	601 ± 250	1806 ± 340	0.18

NT−pro−BNP, N−terminal pro−B−type natriuretic peptide.

## Data Availability

The data that support the findings of this study are available from the corresponding author upon reasonable request.
